# Solving the Differential Biochemical Jacobian from Metabolomics Covariance Data

**DOI:** 10.1371/journal.pone.0092299

**Published:** 2014-04-02

**Authors:** Thomas Nägele, Andrea Mair, Xiaoliang Sun, Lena Fragner, Markus Teige, Wolfram Weckwerth

**Affiliations:** Department of Ecogenomics and Systems Biology, University of Vienna, Vienna, Austria; RIKEN PSC, Japan

## Abstract

High-throughput molecular analysis has become an integral part in organismal systems biology. In contrast, due to a missing systematic linkage of the data with functional and predictive theoretical models of the underlying metabolic network the understanding of the resulting complex data sets is lacking far behind. Here, we present a biomathematical method addressing this problem by using metabolomics data for the inverse calculation of a biochemical Jacobian matrix, thereby linking computer-based genome-scale metabolic reconstruction and *in vivo* metabolic dynamics. The incongruity of metabolome coverage by typical metabolite profiling approaches and genome-scale metabolic reconstruction was solved by the design of superpathways to define a metabolic interaction matrix. A differential biochemical Jacobian was calculated using an approach which links this metabolic interaction matrix and the covariance of metabolomics data satisfying a Lyapunov equation. The predictions of the differential Jacobian from real metabolomic data were found to be correct by testing the corresponding enzymatic activities. Moreover it is demonstrated that the predictions of the biochemical Jacobian matrix allow for the design of parameter optimization strategies for ODE-based kinetic models of the system. The presented concept combines dynamic modelling strategies with large-scale steady state profiling approaches without the explicit knowledge of individual kinetic parameters. In summary, the presented strategy allows for the identification of regulatory key processes in the biochemical network directly from metabolomics data and is a fundamental achievement for the functional interpretation of metabolomics data.

## Introduction

Genome-wide analysis of transcript levels, protein abundance and metabolite concentration has evolved as a central strategy in biology. Numerous studies based on techniques like next-generation sequencing and metabolic phenotyping using liquid chromatography coupled to mass spectrometry (LC-MS) and gas chromatography coupled to mass spectrometry (GC-MS) have significantly contributed to our current knowledge about the molecular organisation of cells, tissues and whole organisms up to the analysis of ecosystems [1], [2]. However, the prediction of dynamic metabolic phenotypes from full genome sequences is still an obstacle [3]. There is an increasing need for methods to systematically analyse and interconnect large datasets from different experiments, in order to uncover global molecular causalities. In recent years, several strategies were developed aiming at the comprehensive analysis of complex data sets. For example, by combining techniques of metabolic profiling and genotyping strong genetic associations for concentrations of metabolites were recently identified to characterize candidate genetic building blocks for lignin content in maize [4]. A different approach of integrating data on transcript levels and metabolite abundance was applied to improve the understanding of global responses to nutritional stresses in *Arabidopsis thaliana* [5]. In yeast, genome-wide approaches to elucidate toxicity mechanism and global stress responses are developed in the emerging transdisciplinary field of Toxicogenomics. It aims at the study of cell response to a given toxicant at the level of the genome, transcriptome, proteome and metabolome. Several studies could already provide an integrative view on how cells interact with their environment [6]. In a biomedical context the combination of metabolite profiling by GC-MS and microarray based gene expression profiling was shown to be a promising approach to provide comprehensive insights into cellular regulation. For example, the analysis of metabolic signatures obtained by an untargeted GC-MS approach was shown to be an important step towards the understanding of human pluripotent stem cells [7].

However, results from many studies also indicated the need to further analyse dynamical behaviour of metabolism in order to understand how a stable metabolic homeostasis can evolve and is sustained under changing environmental conditions [8], [9]. Time-course experiments taking into account diurnal dynamics and circadian rhythms [10], [11] have been proven useful to analyse metabolic regulation and plant-environment interaction. To approximate cellular metabolic networks, techniques of large-scale metabolic network reconstruction based on information about whole genome sequences have been developed and applied to plant metabolism [12]–[14]. These approaches were shown to be able to provide information and allow predictions about metabolic constraints, e.g. total ATP demand for growth and maintenance [13]. Yet, they are severely affected in their predictive power by network gaps and mass-balance errors resulting from incomplete genome annotation and reaction stoichiometry errors [15]. Additional complexity of such large-scale modelling approaches arises from the fact that it is hardly possible to provide sufficient information about reaction kinetics and associated parameters to test and validate predicted rates of metabolite interconversion. In a system of ordinary differential equations (ODEs) describing a metabolic system of interest, the Jacobian matrix, which characterizes the dynamical capabilities of the metabolic system, represents the first-order partial derivative of functions *f_i_* with respect to metabolite concentrations *M_i_* (Eq. 1).
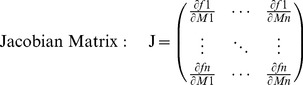
(1)


One central parameter defining these functions in a biological metabolic system is enzyme activity for which experimental data are available only to a limited degree when compared to high-throughput measurements of metabolite content or protein abundance. To overcome this limitation a parametric representation of the Jacobian matrix is possible to allow the characterisation of a metabolic system of interest [16]. In contrast, the inverse calculation of the Jacobian matrix directly refers to covariance data from experimental high-throughput metabolomics data. By this, a characteristic biochemical Jacobian matrix can be estimated, instantaneously linking model structures and experimental high-throughput metabolomics data sets [17], [18]. However, the linkage of genome-scale metabolic reconstruction and metabolomics data is not directly possible because typical profiling strategies such as GC-MS and LC-MS detect only subsets of the whole metabolome. Therefore, we constructed superpathways from a genome-scale metabolic reconstruction which cover the typical set of identified metabolites in a metabolomics approach focusing the central primary leaf metabolism of *Arabidopsis thaliana*. This strategy allowed for the calculation of a differential biochemical Jacobian directly from metabolomics data. We analysed regulatory strategies in primary metabolism of leaves of *Arabidopsis thaliana* induced by conditions of energy deprivation, which is a substantial challenge for a plant due to restricted energy resources and a complex reprogramming of metabolism [19], [20]. Our predictions indicated significant alterations in the pyruvate dehydrogenase complex (PDC) activity which we could validate experimentally. Finally, we applied the obtained information to a parameter optimization strategy.

## Results

### Metabolic Reconstruction of Genome-scale Superpathways and Calculation of the Biochemical Jacobian from Metabolomics Data using a Systematic Mathematical Equation

**Figure 1:**
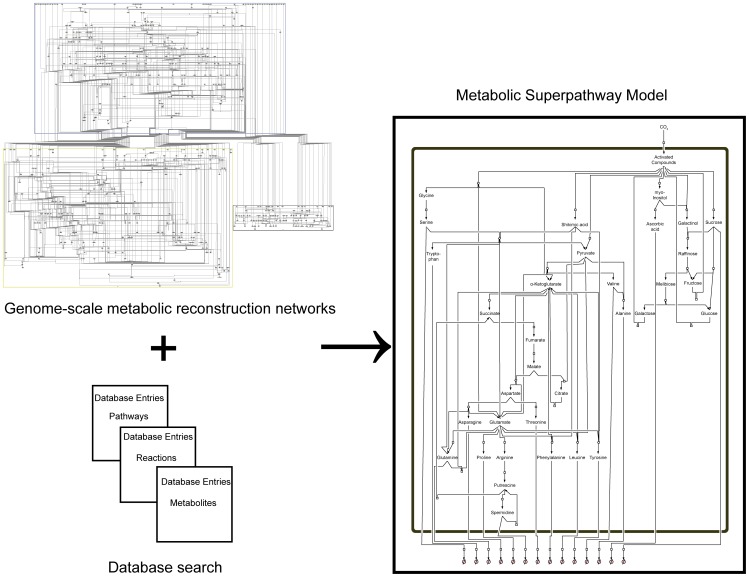
Development of a superpathway model for primary leaf metabolism. The model structure was derived from stoichiometric and biochemical information provided by genome-scale metabolic networks and databases. The model is provided in SBML format in the supplements (Model S1).

Based on the genome-derived stoichiometric matrix of metabolism in *Arabidopsis thaliana* [12]–[14] and database information about metabolic interaction (AraCyc, http://www.arabidopsis.org/biocyc/index.jsp; MetaCyc, http://metacyc.org/) we developed a metabolic superpathway model of leaf primary metabolism in *Arabidopsis thaliana* (Fig. 1). In this simplified model structure, each superpathway represents a summary of underlying reactions directly connecting metabolites which were experimentally accessible. These superpathways were built by removing all metabolic intermediates which were not contained in our experimental GC-MS data set focusing the central leaf primary metabolism of *Arabidopsis thaliana*. Finally, we re-connected all metabolite pools which were left in the model by irreversible reactions. The model contains 49 metabolite pools of which 32 were determined experimentally, and 52 reactions. The model is provided in SBML format (Model S1). The metabolite interaction matrix of the model, which represents a simplified version of the true stoichiometric matrix, was used - together with the experimental covariance data - for the inverse calculation of the Jacobian matrix. Experimental covariance data and the metabolic interaction network were linked by equation 2 which results in entries of the Jacobian matrix [17], [21]:
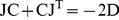
(2)


J represents the Jacobian matrix, C the covariance matrix derived from the experimental data and D is the so-called fluctuation matrix integrating metabolite fluctuations which can be modelled by a Langevin-type equation (Eq. 3):
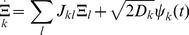
(3)


**Figure 2:**
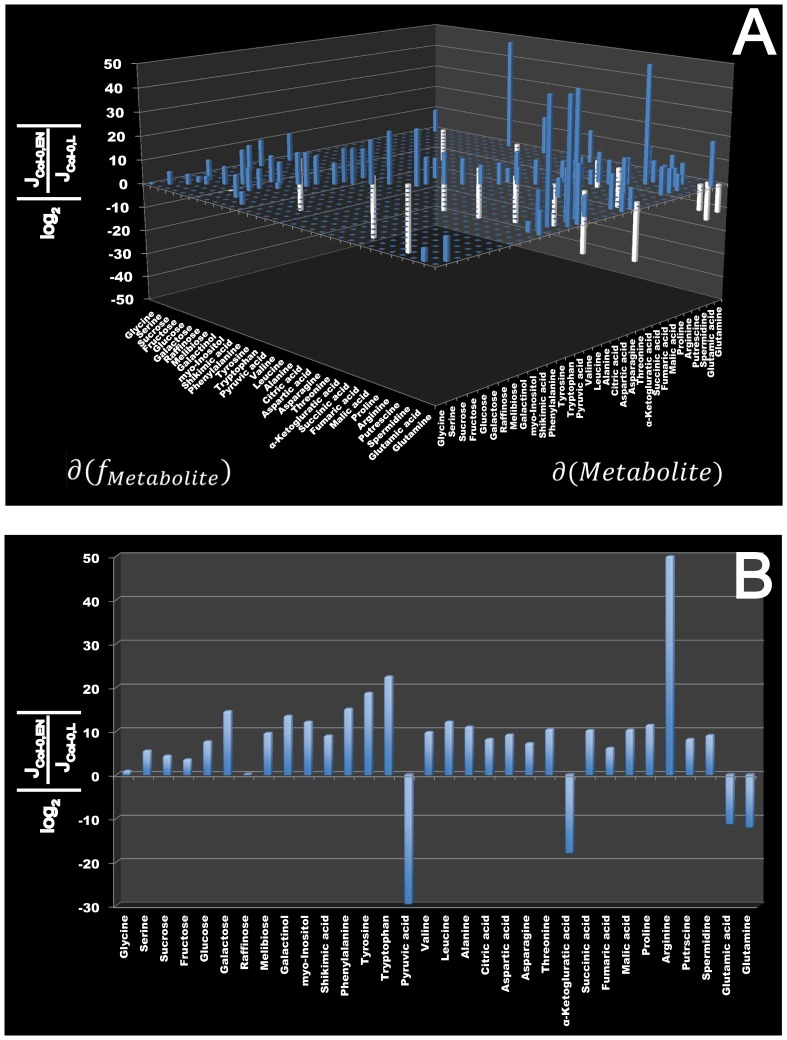
Differential Jacobian of Col-0 under conditions of light and extended night. Bars represent the log_2_-ratio of the entries in the Jacobian matrices of Col-0 under conditions of light (L) and extended night (EN), which were derived from covariance data of metabolomics data sets. Blue colour indicates a ratio >1, i.e. the Jacobian entry of the samples of extended night was higher than under normal light. White colour indicates a ratio <1, i.e. the Jacobian entry of the samples of extended night was lower than under normal light. All entries represent median values of 10^3^ calculations normalised to the square of interquartile distance. (**A**) 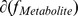 and 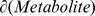 characterize the entries of the Jacobian matrix and refer to equation 1. (**B**) shows the diagonal entries of the Jacobian matrix belonging to the metabolites described on the horizontal axis.

**Figure 3:**
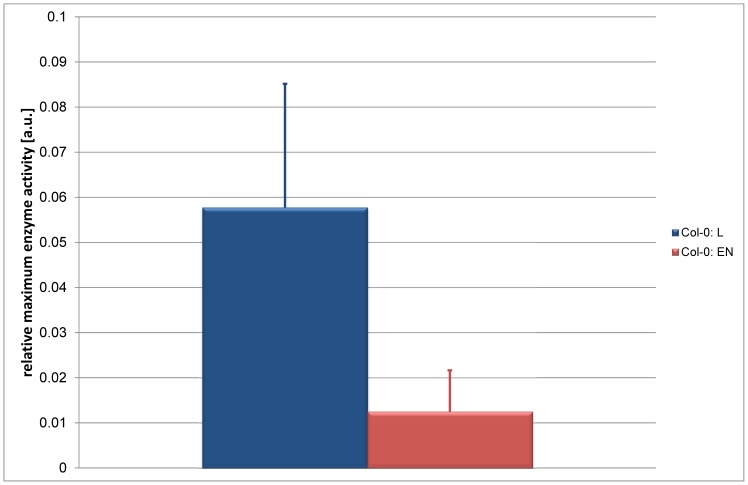
Relative activity of the pyruvate dehydrogenase complex in Col-0 under conditions of light and extended night. Enzyme activity is given in arbitrary units which are normalised to gram fresh weight. The blue bar shows relative activity under normal light condition, the red bar shows activity under condition of extended darkness. The difference of relative activity is significant (p<0.05) and bars represent means ± SD (n  = 5).

Here, time-dependent changes in the matrix of metabolite concentrations Ξ are directly linked to the Gaussian noise function ψ(t). Stationary solutions of (Eq. 3) are linked to the covariance matrix by a corresponding Fokker-Planck equation, finally resulting in (Eq. 2) [21], [22]. Recently, we implemented this equation into a statistical toolbox called COVAIN for the analysis of metabolomics data and demonstrated that the Jacobian can be directly estimated from the data assuming a predefined fluctuation matrix [17]. To solve the inverse Jacobian from the covariance matrix (Eq. 2), a total least square optimization routine was applied [17]. To figure out the impact of the fluctuation matrix on the results of our inverse Jacobian calculation, we repeated the calculations 10^1^, 10^2^, 10^3^, 10^4^ and 1.6×10^4^ times and found the interquartile distance of calculated Jacobian entries to be robust after at least 10^3^ calculations in all data sets (Fig. S1). Medians of 10^3^ calculated Jacobian entries were then normalised to the square of interquartile distances. A differential Jacobian, which describes relative changes between the entries of two Jacobian matrices of different conditions [17], was calculated directly from the metabolomics data of control plants and plants exposed to extended darkness running into the low energy syndrome (LES). The differential Jacobian matrix indicated the most significant difference in the diagonal entry of pyruvic acid (<0) and arginine (>0) between both conditions (Fig. 2 A,B). As the diagonal entries of a Jacobian matrix describes the partial derivative of a metabolite function with respect to the metabolic substrate abundance, an entry <0 indicates a faster change in the metabolite function with respect to a change in the metabolite concentration in the denominator of the differential Jacobian matrix. In contrast, entries >0 indicate a faster change in the numerator. With regard to our data set this means that small changes in cellular levels of pyruvic acid induce a faster change in the pyruvic acid consuming function in control plants than under conditions of extended darkness. Assuming that an enzyme, which follows the Michaelis-Menten kinetics, significantly affects the interconversion of pyruvic acid, we hypothesised that such enzyme activities are changed by conditions of extended darkness. Due to the relative quantification of metabolite abundance in our GC-MS experiment, these predictions are only qualitative indicators of changes in enzyme activities, and absolute quantification is not possible. A prominent metabolic reaction, which interconverts pyruvic acid, is catalysed by the pyruvate dehydrogenase complex (PDC) producing acetyl-coenzyme A. Hence, to validate our model prediction indicating that enzymatic activities are changed between the two conditions, the whole cell activity of PDC was experimentally determined (Fig. 3). Proving the Jacobian-based predictions, PDC activity was found to be significantly higher in samples of control condition than under conditions of extended darkness (p<0.05) and provide a causal explanation for the differential Jacobian entries.

### Application of Results from Inverse Calculation to ODE System Optimization

**Figure 4:**
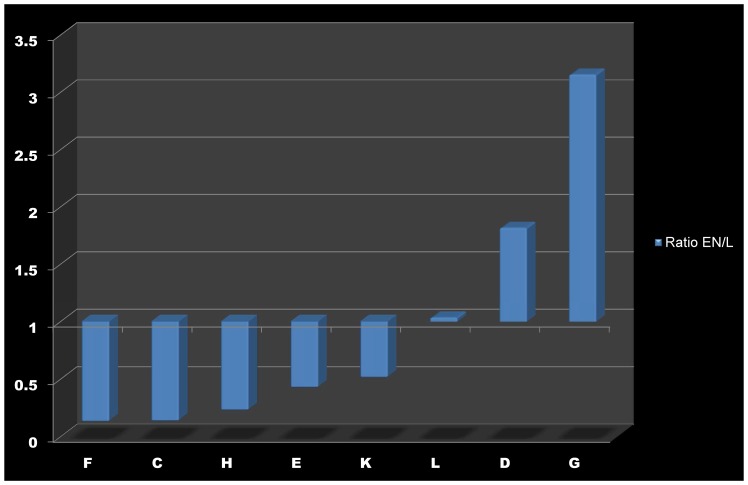
Relative changes of metabolic clusters under light and extended night conditions. Bars indicate the ratios of metabolic clusters from Col-0 under extended night and light conditions. Clusters are named according to the description in the main text (*Results)* and Table S1.

The next step was to analyse whether the finding of differential regulation of pyruvic acid metabolism is sufficient to explain the differences in metabolic homeostases. Metabolic changes were classified according to underlying pathways and measured metabolite abundances were summarized in metabolic clusters (Fig. 4). This resulted in 8 clusters, comprising metabolites belonging to photorespiration (Cluster: C), aromatic amino acids (Cluster: D), sugars (Cluster: E), pyruvic acid (Cluster: F), amino acids derived from pyruvic acid (Cluster: G), TCA intermediates (Cluster: H), amino acids derived from TCA intermediates (Cluster: K) and polyamines (Cluster: L). The composition of the metabolic clusters are described in detail in Table S1.

**Figure 5:**
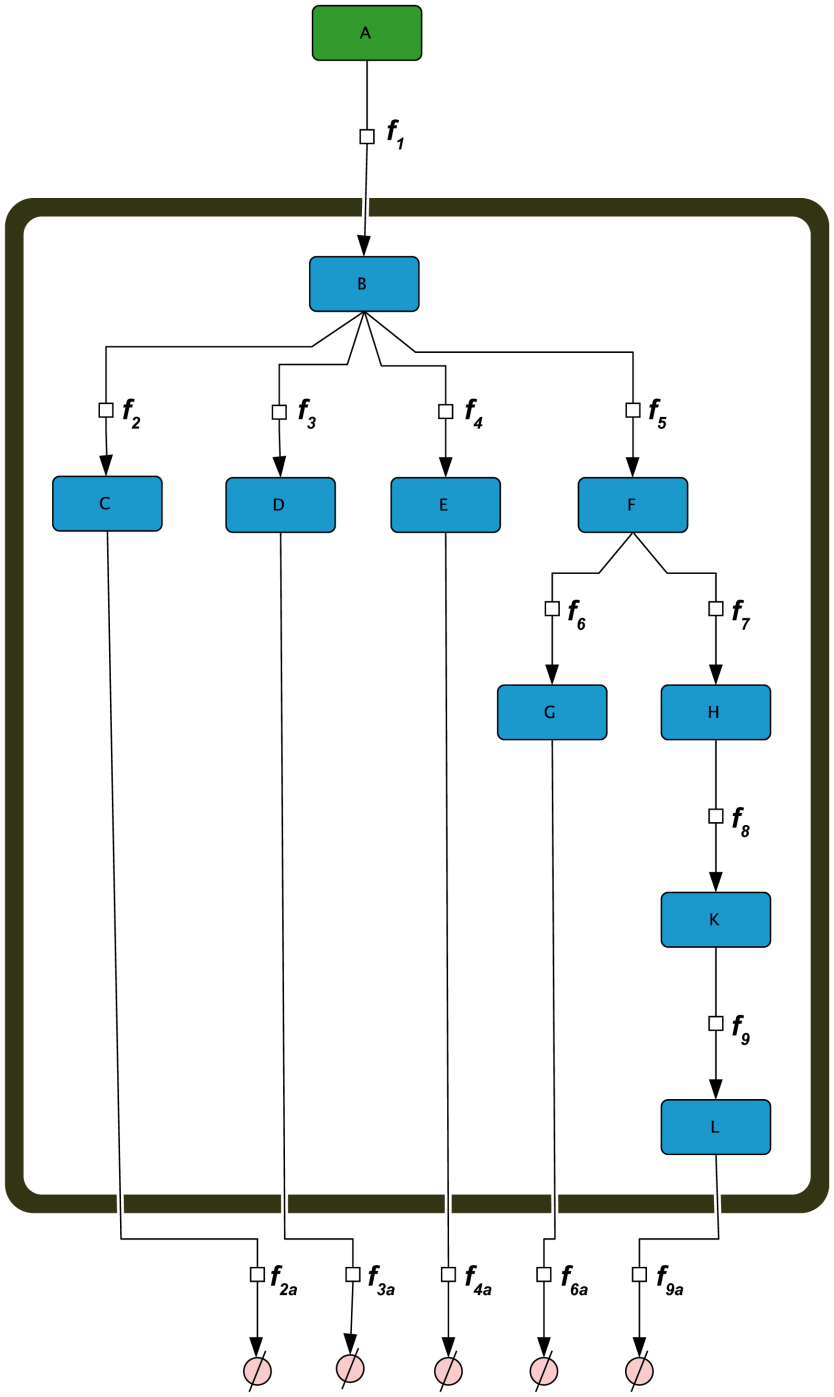
Simplified model structure of the primary metabolism according to metabolic clusters. The model was derived by interconnecting the metabolic clusters by functions of interconversion (*f_i_*). Clusters are named according to the description in the main text (*Results)* and Table S1.

**Figure 6:**
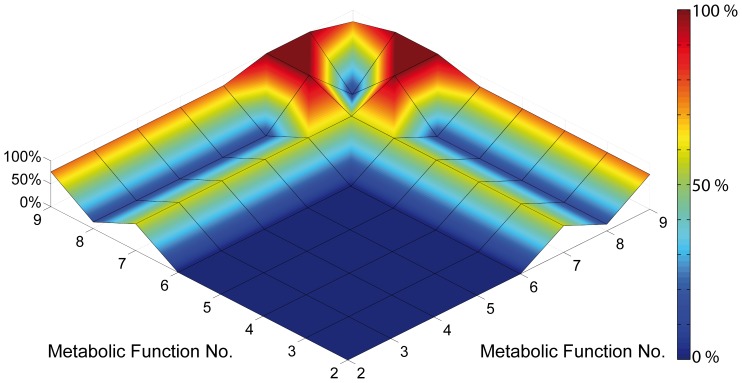
Contribution of metabolite interconversion functions to the best numerical solution of the simplified metabolic network model. Numbers of metabolite interconversion functions are indicated on x- and y-axis. The colour bar indicates the relative decrease of the cost function value, i.e. improvement of solution, when a function or a combination of functions was varied during the optimization process. All cost function values were normalised to the best of all optimization runs (100%). Functions were optimized for the simulation of the metabolic homeostasis of Col-0 under extended darkness.

When transferred to extended night conditions, metabolic clusters C, F and G were affected most significantly when compared to control conditions (Fig. 4). In contrast, levels of polyamines (Cluster L) were least affected. To comprehensively integrate these changes of metabolite levels together with the enzyme activity and the calculated entries of the Jacobian matrices, a simplified model was derived from the superpathway model (Fig. 5). This model was based on ordinary differential equations (ODEs) connecting the metabolic clusters by network functions *f_i_*. These functions *f_i_* represent the abstract summary of metabolite functions, which are defined by parameters, for example like temperature, enzyme abundance or substrate affinity. To test whether the information about relative changes in PDC activity is applicable to the solution of a system of ODEs describing relative levels of metabolite pools, an optimization procedure was applied providing information about the contribution of every single interconversion function *f_i_* to the best solution of the ODEs. The optimization procedure was designed as described in the following: an arbitrary solution of the system of ODEs was calculated allowing for the simulation of the metabolic steady state in Col-0 under normal light conditions resulting in a set of *f_i,Col-0_*. Starting from these identified values of *f_i,Col-0_*,*_L_* a local solution for the system of ODEs was searched which allows the simulation of the metabolic homeostasis of Col-0 under conditions of extended darkness. To evaluate the contribution of each *f_i_* to the best solution, different optimization runs were performed only allowing one or two of the *f_i_* to be varied. For example, in the first optimization run only f_7_, the function which describes the turnover of pyruvic acid, was set as a decision variable in the optimization procedure. Here, the solution only depended on variation of f_7_. In the next run, an additionally function, e.g. f_8_, was defined as a decision variable, too; hence the solution now depended on variation of f_7_ and f_8_. In this way, all combinations of functions were analysed to evaluate the most efficient function combination for yielding the best solution. The landscape showing the relative contribution of each *f_i_* in combination with another function to the best solution indicated the most significant contribution by *f_7_* (turnover of pyruvic acid) and *f_9_* (turnover of amino acids derived from TCA intermediates, polyamine biosynthesis) (Fig. 6).

## Discussion

### The Inverse Calculation of the Differential Jacobian Reveals Changes in Metabolic Functions

Metabolism is organized in a highly complex manner being attributed to a vast variety of interlaced regulatory loops between different levels of molecular organisation, e.g. metabolite-protein interaction, and a high level of cellular compartmentation. This makes it hardly possible to intuitively conclude regulatory strategies from large experimental data sets, which have become a central part of systems biology and are provided by various omics technologies [2], [3]. Computer assisted approaches of mathematical modelling have become an imperative strategy to handle such complex networks to derive as much information as possible about physiological responses, developmental program or cellular function [23]. The results of our study provide evidence that deriving entries of Jacobian matrices from GC-MS data represents an efficient approach to combine high-throughput measurements with mathematical modelling. Such a methodology is central to the research field of systems biology aiming at uncovering biological networks on a global scale [3]. In contrast to the non-limiting conglomeration of mathematical methods, which have become applicable to the research field of systems biology, one of the most limiting steps is the knowledge of model parameters and the validation of model outputs by experimental data. Frequently, this results in a solution space of model simulations with multiple possible flux distributions or parameter sets significantly complicating the determination of the most realistic model output [24]. The systematic linkage of the metabolic solution space defined by metabolomics measurements with the metabolic network structure as represented by our approach exactly tackles this multi-solution problem of structural modelling. To some extent, this limitation can be overcome by the presented method of inverse calculation of the Jacobian matrix. While Jacobian matrices in a metabolic network are analytically derivable as the first-order partial derivative of functions of metabolite concentrations with respect to other metabolite concentrations, this presumes the exact knowledge of the functions of metabolite concentrations. These functions, however, are again composed of various unknown parameters, like enzyme kinetics or thermodynamic constraints. In our approach, the metabolite function does not have to be known explicitly but can indirectly be estimated by the (co-)variance of the experimental data. In the present study, we have verified that the entries of the differential Jacobian matrices indicate relative changes between environmental conditions in rates of metabolite functions depending on enzyme kinetics. Particularly, the diagonal entries of differential Jacobian matrices provide information about how a rate changes with respect to a change in substrate concentration and with respect to the condition. The following example should explain this in more detail: assuming that metabolite function *f_A_* describes the time-dependent concentration change of metabolite A which is predominantly catalysed by an enzyme following the Michaelis-Menten kinetics under conditions C1 and C2, this would result in a characteristic diagonal Jacobian entry *dJ_ii_*:
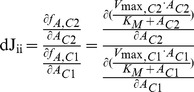
(4)


Based on this equation in context of equation 2 (Eq. 2), metabolomics data can directly be linked to underlying regulatory instances, e.g. enzyme activities. Additionally, due to building a ratio of two Jacobian entries with the same assumption about enzyme kinetics, knowledge about the detailed characteristics of the enzymatic interconversion is not necessary for detecting differential regulation. This significantly reduces the uncertainty about the biochemical reaction network which does not only arise from experimental metabolomics data but also from kinetics and kinetic parameters [25]. By using equation 2, the metabolomics covariance data, and a reconstructed metabolic network of central metabolism in Arabidopsis, Jacobian entries were calculated for the *Arabidopsis thaliana* wildtype Col-0 under control conditions as well as under low energy stress if grown in an extended night period. We found the diagonal Jacobian entries depending on pyruvic acid to be significantly lower in extended darkness. According to our hypothesis of a dominating effect of enzyme kinetics for definition of metabolite functions, the differential Jacobian entries were predicted to be due to a differentially regulated activity of PDC. The PDC enzymatic activity was measured *in vitro* and was found to be significantly higher in the control plants than in the samples of extended darkness. However, the multivariate Gaussian distribution of the stationary solution of the Langevin-type equation significantly affects the fluctuation matrix D [21], [22], [26], and thereby automatically reduces the predictive power of inversely calculated entries of the Jacobian matrix. Additionally, entries of the Jacobian matrix, which are derived from a metabolic interaction matrix, rather represent the collective change in the activities of all enzymes involved. Hence, those entries may only give a reliable prediction about rate limiting steps in the corresponding superpathway, e.g. the activity of PDC. Further studies are now necessary to reduce the noise and to increase the number of reliable predictions, e.g. by integration of more precise information about the metabolic interaction, i.e. protein abundance and thermodynamic constraints.

### Entries of the Biochemical Jacobian Matrix Allow for Prediction of a Parameter Optimization Strategy

The representation of a biochemical system by ODEs has been focused by many approaches of mathematical and kinetic modelling [27]–[29]. While kinetic modelling represents an attractive method to study complex metabolic systems comprehensively, a plethora of information about enzyme kinetics, kinetic parameters, regulation and network topology are needed to be able to reconstruct and predict system dynamics [30]. Based on our finding that the comparison of Jacobian entries predicts changes in enzymatic activity, we tested whether this information is also applicable to solve a system of ODEs of a simplified metabolic network describing groups of metabolites in leaf primary metabolism of *Arabidopsis thaliana*. The central components of a mathematical optimization procedure are decision variables, an objective function and constraints [31]. While decision variables can be varied during the search for the best solution, the objective function indicates the quality of solution and can be minimized or maximized under variation of the decision variables. A prediction of optimization strategies is desirable because, particularly in large-scale metabolic networks but also in time-series models, the computational time increases exponentially with the size of the optimization problem [32]. In our example, we found that, as predicted by the differential Jacobian, the objective function, i.e. the minimal error square of steady state simulation and experimental data, becomes minimal if the reaction of pyruvic acid to TCA intermediates (*f_7_*) is set to be a decision variable. Additionally, the reactions of amino acids to polyamines (*f_9_*) were found to be a pre-requisite for the best solution of the optimization problem. In context of this finding it is interesting to note that the diagonal entry of the differential Jacobian for arginine interconversion was greater than zero which is directly related to *f_9_* as it describes the polyamine biosynthesis. Thus, we provide evidence that inverse calculation of a differential Jacobian based on metabolomics data provides a framework for evaluation of optimization strategies which have to be applied to ODE-based models of complex metabolic networks.

To summarize the findings of the present study, we could demonstrate how metabolomic data from different homeostasis can systematically be linked to large-scale models allowing for the identification of perturbed pathways. Because the calculations of the Jacobian matrix are based on genome-derived stoichiometry of the metabolic network and metabolomics data, this matrix now provides a comprehensive platform for estimating the impact of genetic or environmental impact on regulatory mechanisms in complex biological systems.

## Materials and Methods

### Plant Cultivation

Seeds of *Arabidopsis thaliana* Col-0 were sown on soil, stratified at 4°C for 2 days, and plants were grown in a growth cabinet in a 12 h light/12 h dark cycle. Light intensity was 80 μmol m^−2^s^−1^, and temperature was set to 20/16°C during the light/dark period. Whole rosettes of 35 days old plants were harvested 6 h after light on and after 18 h of darkness, i.e. 6 h of extended darkness. Samples were frozen immediately in liquid nitrogen. Plant material was ground to a fine powder in a ball mill (Retsch, Haan, Germany) and stored at −80°C until further experimental processing.

### Metabolite Extraction, Derivatisation and Analysis by GC-MS

Metabolite extraction and derivatisation was done as described in [18] with minor changes. 1 ml of −20°C cold methanol/chloroform/H_2_O (2,5/1/0,5) was added to 80 mg of plant material. Samples were vortexed, incubated on ice for 8 – 10 min and centrifuged for 4 minutes at 14000 g. 500 μl H_2_O were added to the supernatant, followed by brief vortexing and 2 minutes centrifugation. The polar phase was split equally into 2 aliquots and C13 labeled Sorbitol was added to a final concentration of 10 mg/l as an internal standard. Samples were dried and for derivatisation, the dried pellets were dissolved in 10 μl of a 40 mg ml^−1^ solution of methoxyamine hydrochloride in pyridine by shaking at 30°C for 90 minutes. 40 μl of N-methyl-N-trimethylsilyltrifluoroacetamid (MSTFA), spiked with 60 μl/ml of a mix of even-numbered alkanes, were added and the samples were incubated at 37°C for 30 minutes under constant shaking, followed by 2 minutes of centrifugation at 14000 g. The supernatant was transferred into a glass vial for measurement. GC-MS measurements were carried out on an Agilent 6890 gas chromatograph coupled to a LECO Pegasus 4D GCxGC-TOF mass spectrometer (LECO Corporation, Michigan, USA). Setup, methods and raw data processing were applied as previously described [18] with the following changes: in the GC method, the initial oven temperature was 70°C (held for 1 min), followed by a 9°C/min ramp with 350°C end temperature (held for 8 min). In the MS method, data acquisition rate was 20 spectra/sec with a detector voltage of 1550 V. Acquisition delay was 5.5 minutes and mass range was 40 to 600 m/z. Raw data were processed with the LECO Chroma-TOF software (LECO Corporation, Michigan, USA). Relative metabolite levels for each sample were calculated from the peak areas by dividing by the peak area of the internal standard, subtracting of occurring values in the blank and dividing by the sample fresh weight.

### Inverse Calculation of Jacobian Matrices from Experimental Metabolomics Data

Inverse calculation of the Jacobian matrix was performed applying an algorithm as described previously [17], [18]. Calculations were performed for 10^1^, 10^2^, 10^3^, 10^4^ and 1.6x10^4^ times, and medians as well as interquartiles of calculated Jacobians were determined. Medians were normalised to the square of interquartiles to include fluctuation of calculations in the data interpretation. Differential Jacobian matrices were built as the log_2_-ratio of Jacobian entries from data sets recorded under conditions of light (L) and extended night (EN) (Equation 5):
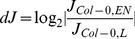
(5)


### Determination of Whole Cell PDC Activity

The activity of PDC in crude extracts of leaf samples of *Arabidopsis thaliana* was assayed by the formation of NADH at 340 nm as described by Yu and co-workers with slight modification [33]. The assay was downscaled to be performed in a 96 well microplate (μClear, Greiner Bio-One GmbH, Germany).

### ODE Programming and Optimization Procedure

Programming and global optimization of ODEs was performed in MATLAB 7.12.0 (R2011a) using the ‘Systems Biology Toolbox 2’ and the ‘SBPD Extension Package’ [34]. The ODE model structure is provided in the Supplementary Information in SBML format (Model S2). The optimization process was performed applying a downhill simplex method in multidimensions. The algorithm is based on section 10.4 in “Numerical Recipes in C” [35], and is implemented in the ‘Systems Biology Toolbox 2’ and the ‘SBPD Extension Package’ [34].

## Supporting Information

Figure S1 **Interquartile distance of calculated Jacobian entries derived from** ***n*** **replicates.** Each interquartile of samples under conditions of light (A) and extended night (B) was normalised to the interquartile distance of 10 replicates.
(PDF)

Table S1 **Metabolomics data.** GC-MS data, mean and cluster-analysis results.
(XLSX)

Model S1 **Metabolic reconstruction of genome-scale superpathways adapted to metabolite profiling data for Arabidopsis thaliana (see Materials and Methods).**
(XML)

Model S2 **ODE model global optimization procedures (see Materials and Methods).**
(XML)
