# Supplementary figures and images for: Solving the Differential Biochemical Jacobian from Metabolomics Covariance Data
Source: PLoS One. 2014 Apr 2;9(4):e92299. doi: 10.1371/journal.pone.0092299 (PMC3977476; doi:10.1371/journal.pone.0092299)

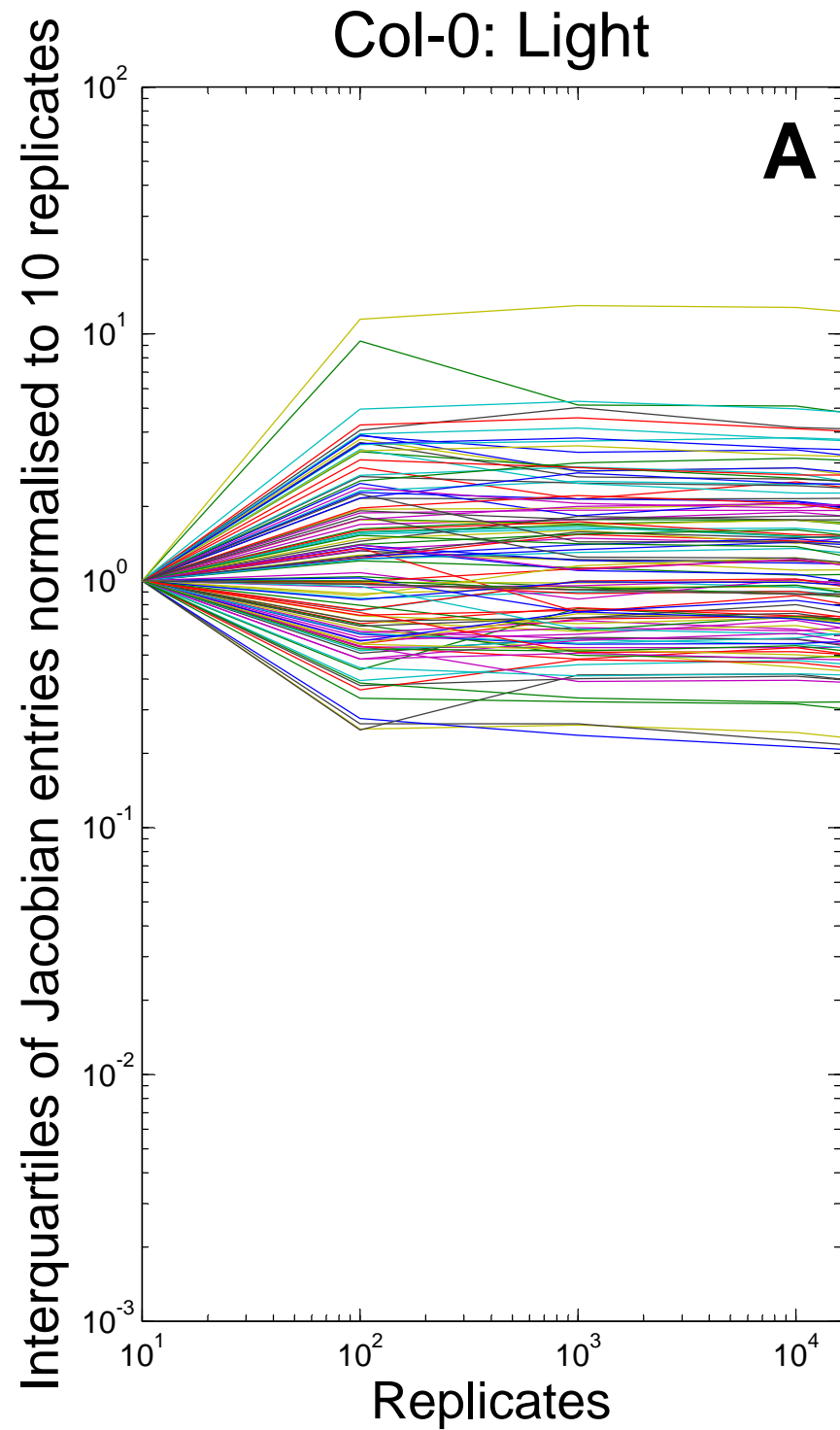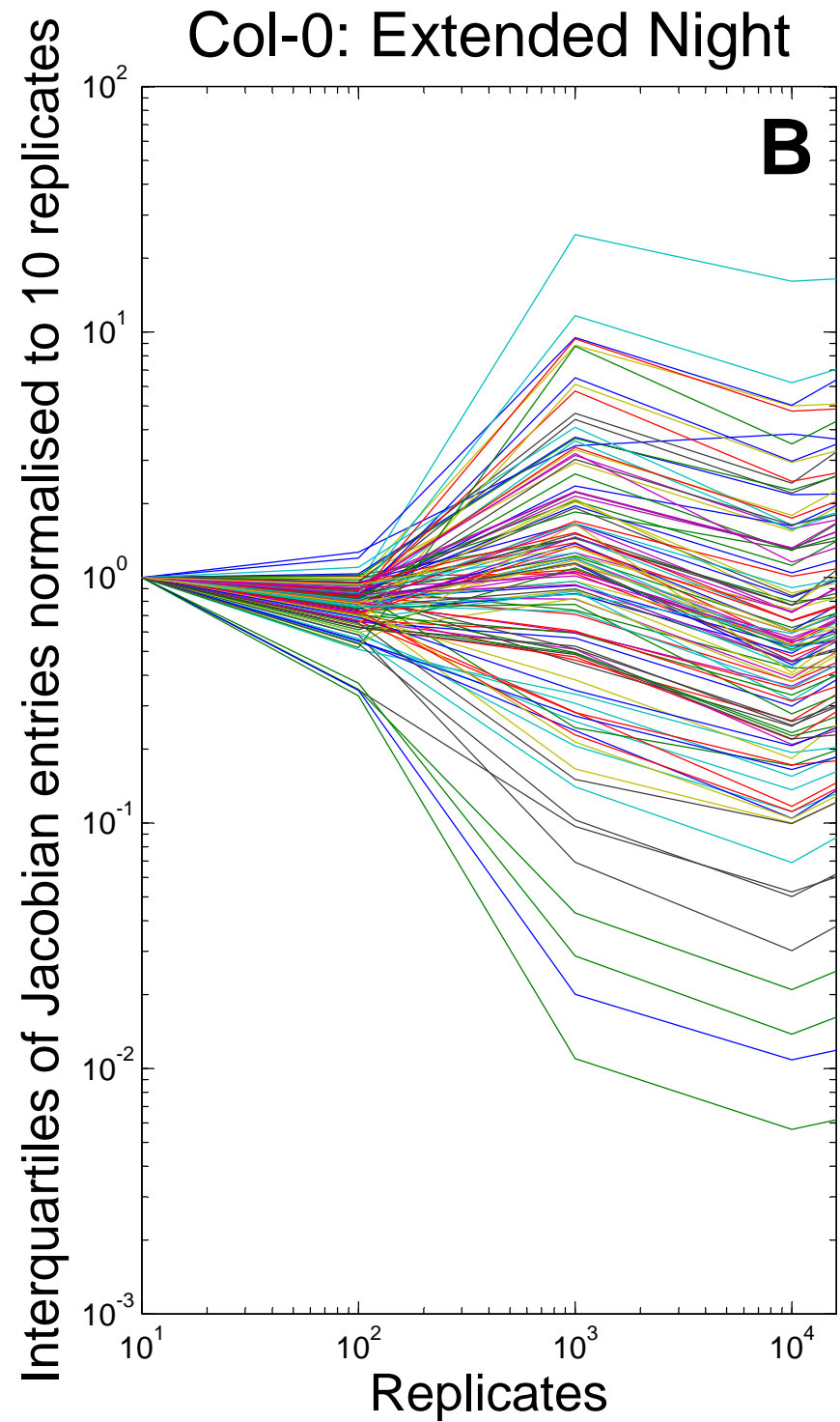

Supplement: Figure S1 — Interquartile distance of calculated Jacobian entries derived from n replicates. Each interquartile of samples under conditions of light (A) and extended night (B) was normalised to the interquartile distance of 10 replicates. (PDF) [file pone.0092299.s001.pdf]
